# Astro‐ecology? Shifting the interdisciplinary collaboration paradigm

**DOI:** 10.1002/ece3.4455

**Published:** 2018-08-31

**Authors:** Jessie C. Buettel, Barry W. Brook, Andrew Cole, John Dickey, Emily J. Flies

**Affiliations:** ^1^ School of Biological Sciences University of Tasmania Hobart Tasmania; ^2^ ARC Centre of Excellence for Australian Biodiversity and Heritage Sandy Bay Tasmania; ^3^ School of Physical Sciences University of Tasmania Hobart Tasmania

**Keywords:** collaboration, ecology, forests, interdisciplinary, transdisciplinary

## Abstract

We present a case study whereby ecological research on fallen trees in forest plots was advanced by a collaboration with astronomers working on the vector fields of stars and gas, and we propose a framework by which such novel collaborations can progress.

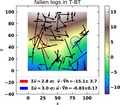

The transfer of methods and analytical approaches across disciplinary boundaries (termed “*migration*” by Geertz, 1980) has become an important feature of scientific studies (Klein, 1999), often fostering the type of innovation and novelty that defines exemplary research (Loeb, 2016). Although many obstacles confront researchers seeking to pursue an interdisciplinary program (Rhoten & Parker, 2004), such collaborations can offer fresh perspectives, adaptable techniques, unity of knowledge, and encouragement of out‐of‐the‐box thinking. Ecology is a discipline that has always benefited from wide‐ranging knowledge due to its inherent complexity (e.g., studies across multiple scales), and indeed, interdisciplinary collaborations are becoming increasingly important as we seek to anticipate, mitigate, and grapple with the impacts of global change on biodiversity and ecosystems (Coreau, Pinay, Thompson, Cheptou, & Mermet, 2009). However, today's interdisciplinary alliances are often formed out of a sense of necessity or obligation (provoked by the need to solve a common problem) rather than preemptively, out of a desire to better understand a complicated process or specific question that may indeed be discipline specific. As such, they might not be reaching their full potential for directed research.

The goal of this essay is to showcase a recent example of an interdisciplinary collaboration that yielded novel insights into the analysis and interpretation of ecological data. The ecology researchers were grappling with a simple, tangible question: “why do trees fall in a given direction?” At the root of this inquiry was a need to understand historic processes (the past death of trees in a forest) from current patterns (the spatial arrangement of logs on the forest floor). The ecologists recognized that this problem (vectors arranged in a multidimensional plane with a heterogenous surface) is encountered frequently by astronomers, and so they reached across the disciplinary gulf for input. Here, we describe the framework under which we successfully worked together, and use this case study to offer guidance on how other researchers might attempt similar ways of doing interdisciplinary research. We show how such collaborations can enrich the quality of the thinking and science produced by researchers.

## 1 THE CURRENT INTERDISCIPLINARY LANDSCAPE IN ECOLOGY

Tackling broad environmental issues typically requires collaboration across physical, biological, social, and statistical sciences (Moss et al., 2010). The cultural context and framing of these issues shapes whether—and why—they are perceived to be important and so underpins the actions society is likely to take (Hackmann, Moser, & St. Clair, 2014). We acknowledge that questions like “How to make the management of the Great Barrier Reef sustainable?” (Hughes et al., 2007) require interdisciplinary representation, and in such cases, it is relatively straightforward to identify what expertise will be needed and what role each researcher will perform. In this example, members would include policy analysts (to understand the managerial and political landscape), scientists (who can present the current state of technical knowledge on the issue and evaluate the probability of success of a given policy), economists (to frame and analyze the cost–benefits of each scenario), and social scientists (to reflect on impacts to human well‐being and challenges in securing a “social license” from public and private stakeholders). The a priori expectation is that this type of interdisciplinary effort (be it research or application) will be logistically and intellectually challenging and will potentially expose participants to irresolvable differences of viewpoint.

However, many have argued that all of the easy questions in ecology have been answered and all that are left are the “wicked” problems: those rife with the interdependencies, uncertainties, and circularities that often characterize ecological systems (Churchman, 1967). Such problems have always required interdisciplinary effort, but thus far, too many of these “wicked” problems are yet to be resolved, or indeed, mitigated (e.g., climate change). Cornell law (Lazarus, 2009) has coined these as “super wicked problems”; the longer the problem takes to be addressed/solved, the harder it will be to do so. Interdisciplinary collaborations can unlock the imagination and bring much‐needed perspective and novel solutions to these diabolical problems (Harris, Brown, & Russell, 2010). We show that it does not necessarily take an inherently interdisciplinary question to benefit from cross‐disciplinary collaborations in ecology. Simply, opening a dialogue with researchers in disparate disciplines about hypotheses may be the crucial first step. As such, we suggest an alternative to the classical interdisciplinary approach: one that begins with a discipline‐specific dataset and a restricted question that involves seeking insight from other fields which might face analogous problems or patterns. That is, flip the common approach to interdisciplinarity.

Many areas of science face logistic, inferential, economic, and ethical constraints similar to ecology. For example, experimentally answering questions in ecology (e.g., how a forest will respond to an invasive species or wildfire) faces similar obstacles to immunology (e.g., how the human immune system will respond to a novel virus). Methods are advancing rapidly in each field to deal with these constraints, but their application is often highly specific. Most researchers recognize the challenge of keeping up with the rapid progression of analytical and technological advances in one's own field, let alone the advances in other disciplines. Collaborating with experts across the range of disciplines allows a quick peek into the advances of other fields and the opportunity to adapt such techniques to the challenging and “wicked” problems of ecology. A famous example of the success of interdisciplinary thinking is (the somewhat clichéd) reference to theoretical physicist Richard Feynman taking a year off from his research on quantum mechanics to work on viruses in a Caltech genetics laboratory (Haynie, 2007). But what could an ecologist gain from consulting with, for instance, an astrophysicist? Such interactions may appear as frivolous, with little apparent common ground, but can actually be a quite useful and broadening experience for all involved.

**Figure 1:**
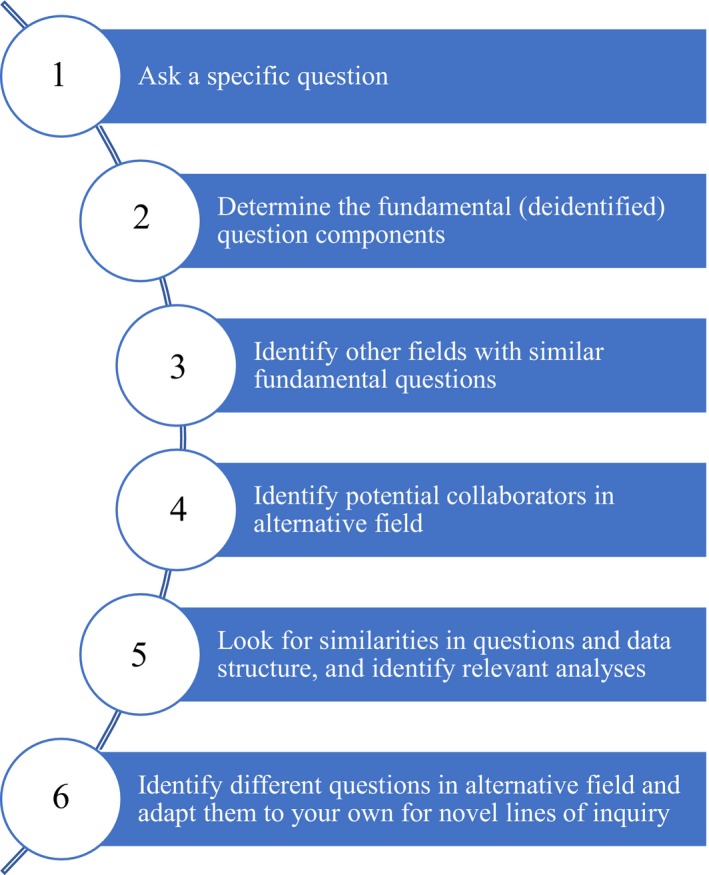
Conceptual outline of how our interdisciplinary collaboration, “Astro‐ecology,” was successful. Step 2 requires the question to be deidentified (“unpacked” into its fundamental process‐ or data‐based components). It then proceeds by asking “what would another scientist do, and what tools would they use if faced with identifying such patterns (Step 3)?” Steps 1–4 allow for a focus on looking for similarities and/or differences in data structure and modeling/analysis (Step 5). This process is also reciprocal (Step 6)—for example, ecologists may apply their expertise in working with complex systems to understanding stellar phenomenon

Any interdisciplinary collaboration should begin with a well‐considered and tightly specified question (Figure 1). Having only vague ideas risks wasted time and decreases the likelihood that the disparate researchers will be able to frame it successfully against their discipline‐specific conceptual models (Brandt et al., 2013). In short, such work requires the question to then be refined, or “unpacked” into its fundamental (process‐ or data‐based) components. This process might best start from a precompiled dataset and a targeted problem that is discipline specific. It can then proceed by asking “what would another scientist do, and what tools would they use if faced with identifying such patterns?” An advantage of breaking down the question is that it allows you to look for similarities and/or differences in data structure and modeling/analysis in other fields. Indeed, the key to the collaborative success in the following example was its prior investment (a graduate student's project, funding, and use of a detailed dataset), and tractable question.

## 2 AN ASTRO‐ECOLOGICAL CASE STUDY

Astronomy actually faces many problems that are akin to those in ecology (Keddy, 1994). To understand the causes and consequences of change at cosmic scales, Earth‐bound observers are obliged to use space‐for‐time substitution to infer dynamical processes and test theoretical models (including understanding limits and exceptions of those models). Astronomers also study the aggregate attributes of statistical populations of “static” entities, because processes at galactic and larger scales operate on timescales that far exceed the life span of any individual observer. The data collected by telescopes represents a snapshot of events that take aeons to unfold. By collating many such “instantaneous” measures across space, researchers can build probable sequences of events that link the snapshots into a coherent whole. They can then test hypotheses to describe the forces and circumstances that trigger, govern, and terminate phenomena on the largest observable scales of the universe.

This setting is analogous to many of the problems faced in ecology, evolution, and environmental science. Indeed, comparisons can be drawn between entities within an old‐growth forest and those of a galaxy (e.g., trees as stars, species as spectral classes, ecological succession as stellar evolution, wildfire as supernova shockwaves, treefall gaps as interstellar clouds). Ecology–astronomy collaborations might therefore unveil the rules that shape the formation, maintenance, and resilience of complex systems like forests or galaxies.

**Figure 2:**
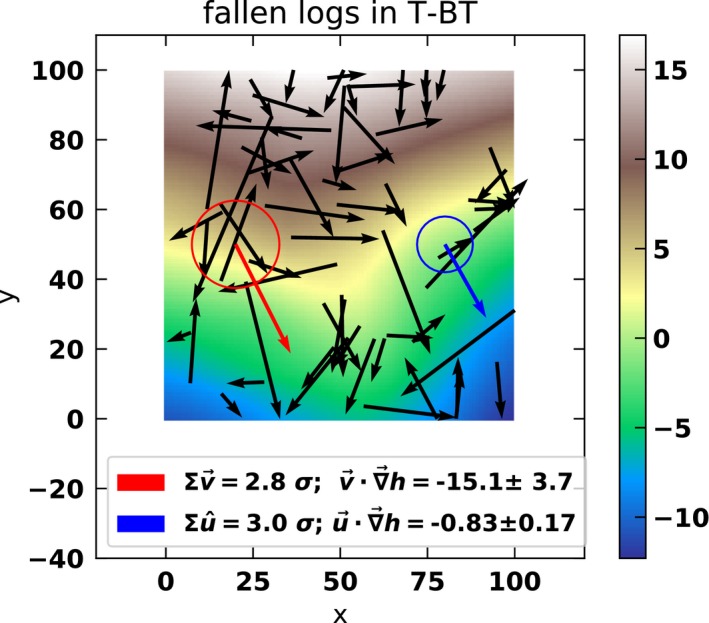
Example of a one‐hectare (100 × 100 m) plot (named “T‐BT”) located in the tall eucalypt forests of Tasmania. The background colors are gradient from low (light blue) to high (red–white). The black lines are the measured treefall events, including length and direction (black arrowhead). The red and blue arrows show the average direction of all logs as indicated by vector analysis, a method borrowed from the field of astrophysics

The challenge for interdisciplinary inquiry in ecology is to bring together these different fields and their ideas or toolkits in such a way that can inform each other synergistically, rather than competing for legitimacy. In this case study, the ecology researchers had developed a large dataset of spatial locations and attributes of trees (including fallen trees) in various “snapshot” plots measured across the tall eucalypt forests of Australia (Wood, Prior, Stephens, & Bowman, 2015). Through discussions with the astronomers, they were able to adapt a method developed to infer the relative redshift (or blueshift) of stars from their spectral lines (current speed, trajectory, and surrounding gravitational fields). The researchers substituted trees for stars or gas clouds, direction of treefall for spatial trajectory, and topographic gradient for gravitational fields and found that trees do indeed fall down slope, but also that there is a threshold of slope and size that most profoundly influences the direction (Figure 2).

This collaboration showed that one discipline could apply methods to a “foreign” yet superficially similar dataset generated by another discipline. But more than that, it quickly became evident that a fertilization of new ideas blossomed as a result of working on a well‐defined problem, and as a consequence, new lines of inquiry were opened to researchers in both fields. The ecologists did not strictly *require* an interdisciplinary collaboration (there are many well‐developed methods of spatial data analysis and modeling of fiber‐process patterns in forest ecology). However, pursuing this approach led to an arguably more informative (and certainly novel) analysis and spawned a plethora of ideas for further future research. Likewise, for an astronomical dataset (e.g., one that consists of spatial point patterns, as stars or galaxies that have distinct features that can be analogous to traits of tree species such as size, age, and evolutionary stage), ecologists might readily lend their expertise and analytical techniques.

## 3 CHALLENGES AND LESSONS LEARNED?

Despite the many benefits, “interdisciplinarity runs counter to traditional ways of thinking, behaving, planning and budgeting in many institutions” (Klein, 1999). Success in academia is (unfortunately) measured principally by number of publications and size of grants awarded, both of which are strongly constrained by “disciplinarity” (i.e., higher grant and publication success typically follows when one is considered an expert within a single discipline; Metzger & Zare, 1999). For example, as Bromham et al. (2016) showed, in a study of 18,500 Australian Research Council's Discovery Programme proposals across 5 years (these are similar to U.S. National Science Foundation Program grants), the greater the degree of interdisciplinarity in a proposal, the lower was the probability of it being funded. This negative outlook on interdisciplinary research stymies creativity, collaboration, and “out‐of‐the‐box” thinking, despite these collaborations being recognized as what many scientific fields need in order to progress (Brockman, 2012). Interdisciplinary research often has broad societal and economic impacts, but these take time to materialize and are not captured by citations (van Noorden, 2015). Fortunately, there are moves to increase the recognition of alternative research outputs—such as educational outcomes, and application to science policy or management—to potentially defray the career costs of interdisciplinary efforts (Goring et al., 2014). As in our example, answering small, focused questions with an interdisciplinary team can effectively move ecology (and science in general) toward comprehensive knowledge and potentially greater societal impacts.

Here, we have highlighted the idea that one discipline can apply methods that speak innovatively to a dataset generated by another discipline. More than that, in our experience, new ideas quickly blossomed and become interwoven with many aspects of the students’ dissertation (lead author JCB). Because a tractable problem was brought to the transdisciplinary table, interactions grew organically from the bottom‐up, rather than being imposed from an extensive top‐down framework. A diversity of interactions evolved: Discussions gave way to debates which resulted in invited lectures in each other's classes. Plots turned into models, and eventually informative analyses and coauthored papers. The potential for such interdisciplinary exchange of ideas and approaches is on offer to a suite of disciplines, not only those mentioned in our example. Even if such collaborations fail to result in joint projects and publications, those involved still have the joy of exploring novel ideas and analyses—a major underpinning of innovation and “out‐of‐the‐box” thinking. In short, there is little to lose, and possibly much to gain, by testing the interdisciplinary waters.

## AUTHOR CONTRIBUTIONS

All authors contributed substantially to the conception or design of the work, or the acquisition, analysis, or interpretation of data for the work. All coauthors assisted in drafting the work or revising it critically for important intellectual content. All authors have consented that this final version to be published and agree to be accountable for all aspects of the work in ensuring that questions related to the accuracy or integrity of any part of the work are appropriately investigated and resolved.

## DATA ARCHIVING

The data supporting the results in this manuscript will be archived in a publicly accessible repository upon acceptance.
